# Association between Depressive Symptoms and Adherence to the Mediterranean Diet in Nursing Students

**DOI:** 10.3390/nu15143158

**Published:** 2023-07-15

**Authors:** Vanessa Ibáñez-del Valle, Rut Navarro-Martínez, Omar Cauli

**Affiliations:** 1 Department of Nursing, Faculty of Nursing and Podiatry, University of Valencia, 46010 Valencia, Spain; maria.v.ibanez@uv.es (V.I.-d.V.); omar.cauli@uv.es (O.C.); 2 Frailty and Cognitive Impairment Organized Group (FROG), University of Valencia, 46010 Valencia, Spain; 3 Chair of Active Ageing, University of Valencia, 4610 Valencia, Spain

**Keywords:** Mediterranean diet, students, anxiety, depression

## Abstract

With university admission, there are major changes in students’ daily habits that can lead to mental health problems. In this respect, adherence to a healthy diet, such as the Mediterranean diet (MD), can be very beneficial. The present study examines the associations between adherence to the MD and mental health among Spanish nursing students (n = 289). Sociodemographic data and life habits were collected electronically using a self-administered questionnaire. The participants also completed the Mediterranean Diet Adherence Screener (MEDAS-14) and the Goldberg Anxiety and Depression Scale (GADS). The percentage of anxiety and depression symptoms was high: 45.3% (n = 131) and 46.4% (n = 134), respectively. Only 35.6% reported good adherence to the MD (score ≥ 9). The statistical analysis showed poor adherence to the MD to be significantly and positively associated with depressive symptoms (*p* = 0.013) and the total score on the GADS (*p* = 0.039). A multivariable regression model analysis identified the depression subscale score as a predictor variable, with a mean risk of low adherence to the MD being 0.803 times (95%CI: 0.666–0.968, *p* = 0.021) among participants with greater depressive symptoms. These results support the implementation of prevention programs in universities focused on health and mental health issues.

## 1. Introduction

The Mediterranean diet (MD) is a centuries-old tradition that provides wellness and helps maintain excellent health [1]. This diet was observed in Greece and southern Italy during the 1960s and was first defined as a dietary pattern characterized by a diet low in saturated fats and rich in vegetable oils [2]. Different associations, such as the Diet Foundation [3] and Oldway’s Preservation and Rediscover Goodness Oldway’s Cultural Food Traditions, proposed three MD pyramids that recommend the following: (a) olive oil, vegetables, fish, bread, and cereals > 6 servings/day; eggs, legumes, and nuts 3–4 servings/day; (b) olive oil at each meal, vegetables, fruits, fish, legumes, and cereals ≥ 2 servings/day; and (c) olive oil, vegetables, fish, legumes, cereals, and bread at each meal. In addition, the MD may also include moderate consumption of dairy products and wine during main courses and low consumption of red meat [4].

The MD has shown a protective effect against cardiovascular disease [5,6] and has been associated with a lower risk of diseases such as cancer [7,8,9] and diabetes [8,10,11]. Despite these benefits, the traditional DM is now progressively deteriorating because of the effects of the modern era, such as the globalization of food production and consumption, the widespread diffusion of a Western-type economy, and urban and technology-driven culture. In this context, non-communicable diseases are becoming more and more frequent [12], and among these, mental health problems stand out. From 1990 to 2013, years of life lost due to disability caused by mental illness and substance abuse increased by 45%, and depressive disorders increased by 53.4% [13]. These data imply high morbidity worldwide, with significant personal, psychosocial, and economic repercussions.

The World Health Organization (WHO) has defined positive mental health as “a state of well-being, both emotional and psychological, where the individual recognizes his or her potentials, adapts to the natural pressures of life, leads productive and supportive work and meets the demands of daily life” [14,15]. Anxiety and depression are common and represent a growing global health and social problem [14,16,17]. Young people are at increased risk for mental illness during early adulthood [18], and many mental health problems begin between the ages of 17 and 24 years [19]. Among health science students, such as medical students, the prevalence of depressive illness ranges from 9% to 55% [20]. In addition, research findings show that fear of depression and suicide has intensified during the coronavirus pandemic (COVID-19), especially among adolescent girls [21]. According to a report from the National Statistics Institute (INE), the leading causes of death in Spain in 2021 among people aged 15–39 years were external causes (43.3% of the total), with an increase of 8.5% over 2020. Among these, suicide remained the leading cause of external death, with 4003 deaths, representing 1.6% more than in 2020 [22]. These are very alarming data that demonstrate the need to establish preventive measures, particularly among university students. Indeed, university students are “a special group of people going through a critical period of transition from adolescence to adulthood that can be one of the most stressful times in a person’s life” [23]. Entering university life means facing important challenges that affect social, personal, and intellectual development, and these changes in daily habits can have an impact upon psychological and physical health [24]. In addition, there are other stressors, such as increased independence or shared housing. Therefore, mental health problems among college students are of increasing concern. Among these problems, the most common are depression and anxiety [25,26].

According to several studies, adherence to the MD may decrease the risk of cancer and cardiovascular disease [4]. However, the possible effects on mental health have not yet been adequately investigated [27]. Two clinical trials [12,28] suggested significant beneficial effects of DM use in people with depressive symptomatology, reducing symptomatology and improving recovery rates. 

Therefore, the main aim of our study was to analyze adherence to the MD in association with anxiety and depressive symptoms in university students and the role of sociodemographic factors in these associations.

## 2. Materials and Methods

### 2.1. Study Design and Population

A cross-sectional survey was conducted between October 2022 and March 2023 among students of nursing or podiatry degree programs at the University of Valencia (Valencia, Spain). The exclusion criteria were individuals under 17 years of age and unwillingness to participate. 

Considering that the accessible population of nursing students was 901, a sample size of 257 randomly selected subjects suffices for estimation with a 95% confidence level and a precision of ±5 percentage units, considering a prevalence of depressive symptoms in university students of around 30% according to data published in Spain [29] and in other countries [30]. A replacement rate of 10% was anticipated. This research was approved by the Human Research Ethics Committee of the University of Valencia (Spain) (procedure number 2298864, 13 October 2022). Participants received information about the study before accessing the data collection instrument and explicitly expressed their desire to participate. The anonymity and confidentiality of the data were maintained by setting up an online survey. 

### 2.2. Information Collection

The survey included ad hoc questions addressing sociodemographic variables (age, gender, marital status, cohabitation, and employment status) and health data (chronic diseases and perceived health status). Perceived health status refers to the individual self-assessment of personal health and has been considered a good predictor of actual health status [31]; it was assessed using a visual analog scale from 1 to 5 points, with the highest score corresponding to a better state of health. Self-perceived health (SPH) is included in one of the 6 groups of determinants of the Active Aging model proposed by the WHO [32]. 

In addition, lifestyle-related data such as weight, height, alcohol consumption, and smoking were also collected. To assess the intake of beverages capable of influencing mental health problems, we asked the participants to report their average daily intake of cups of coffee (black coffee, latte, American coffee), cups of tea, and cola or other energy drinks. Alcohol consumption was assessed using the Alcohol Use Disorders Identification Test-Concise (AUDIT-C). It is a brief alcohol screening instrument that reliably detects persons who have active alcohol use disorders (including alcohol abuse or dependence) or who are at-risk drinkers. It consists of three questions and is scored on a scale of 0–12 [33]. Each AUDIT-C question has 5 response alternatives that are scored from 0 points to 4 points. A score of 4 or more is considered positive for men and optimal for identifying hazardous drinking or active alcohol use disorders. For women, a score of 3 or more is considered positive.

Students were asked to provide information about their college courses and studies. Each student was also asked to report the current grade point average (GPA) of his or her academic record to assess academic performance. The Mediterranean Diet Adherence Screener (MEDAS-14) [34] was used to assess the level of adherence to the DM. Depressive and anxiety symptoms were assessed with the Goldberg Anxiety and Depression Scale (GADS) [35].

Data were collected electronically using a self-administered questionnaire designed ad hoc with Google Forms. The link to access the survey was distributed to students through the Official Platform of Teaching Support Resources (Virtual Classroom) of the University of Valencia. The questionnaire was answered anonymously in the classroom and with the supervision of the researchers. Survey participants did not receive incentives. 

### 2.3. Depressive and Anxiety Symptoms

To assess the level of anxiety or depression, we used the Goldberg Anxiety and Depression Scale, which has been validated in the Spanish language [36]. This is a questionnaire that discriminates between the diagnosis of anxiety and depression and measures their retrospective intensities. It consists of two subscales: one for anxiety and one for depression. Each of the subscales is composed of 9 dichotomous response items (YES or NO) to determine whether the subject has had any of the indicated symptoms in the last two weeks. The cut-off points are 4 or more items or affirmative responses for the anxiety scale and 2 or more for the depression scale. The higher the score, the greater the severity of the problem, with a maximum possible score of 9 points for each of the subscales.

### 2.4. Adherence to the Mediterranean Diet

Adherence to the MD was assessed by means of an online classroom survey using the MEDAS-14 questionnaire under the supervision of the investigators. This is a short questionnaire that can be easily used to quantitatively estimate the level of adherence to cardioprotective Mediterranean diets. It consists of 14 short questions intended to provide information on adherence to the MD pattern. This instrument can be very useful in clinical practice, as it allows the rapid estimation of adherence to the MD [37]. The MEDAS-14 was used with the Spanish population by Estruch et al. [5] in the Prevention with Mediterranean Diet (PREDIMED) study conducted from October 2003 to January 2009 with 7146 participants. Schröder et al. [37] evaluated the relative and construct validity of the 14-item Mediterranean Diet Adherence Screener (MEDAS-14) used in the PREDIMED study, a primary prevention trial with nutritional intervention. The questionnaires available to measure adherence to the Mediterranean diet include the assessment of moderate alcohol consumption. However, the dietary pattern of the MD regarding alcohol has been revised regarding wine intake [38]. The latest DM food pyramid does not address a specific amount for alcohol consumption, suggesting the opposite: “wine in moderation and respecting social beliefs”. Thus, in our research, the updated version of the MD pyramid was considered, and the question on wine intake was excluded (question number 8: “Do you consume wine? How much do you consume per week?”). In addition, for some population groups, such as young adults and pre-conception and pregnant women, alcohol intake is not indicated at all. For these reasons, previous studies such as Ruggeri et al. [39] excluded wine consumption when measuring adherence to the MD to apply these instruments in all population groups to make comparisons among different populations. It is a questionnaire consisting of 14 questions, and each question is scored between 0 and 1 point. The score reveals good adherence to the DM when it is ≥9 points and poor adherence when it is <9 points.

### 2.5. Statistical Analysis

Data analysis was carried out with the SPSS version 26 statistical package (IBM Corp., Armonk, NY, USA) licensed to the University of Valencia. The characteristics of the participants are presented using descriptive statistics. Using a multiple logistic regression model, the associations between the independent variable (adherence to DM) and the dependent variables (mental health, smoking, and body mass index (BMI)) were determined. Statistical significance was considered at *p* < 0.05.

## 3. Results

### 3.1. Characteristics of the Study Sample

A total of 289 students of the nursing degree program (250 women and 39 men) with an average age of 20.60 (±2.56) years (range 17–30) completed the online survey. Almost all the students were single (n = 276), childless (n = 288), and lived mainly with their nuclear family (n = 198). Likewise, 82.4% of the students (n = 238) reported not suffering from any chronic diseases, 74% (n = 214) had normal body weight according to their BMI (kg/m^2^), and—with an average score of 7.79 (±1.29) points out of 10 points—showed a good self-perception of their state of health. Of the total, 13 students reported using psychotropic drugs daily. The characteristics of the sample are presented in Table 1 below.

Continuous variables are expressed as the mean ± standard deviation (SD); categorical variables are expressed as frequency (percentage) over the total sample.

### 3.2. Consumption of Coffee, Cola, Tea, and Alcohol and Smoking

A total of 61.9% of the participants (n = 179) reported consuming stimulant drinks daily, with an average daily consumption of 1.81 (±0.91) cups of coffee, 1.47 (±0.73) cups of cola, and 1.29 (±0.50) cups of tea. In turn, 92% (n = 266) reported not smoking, and 58.1% (n = 121) reported alcohol consumption. Of the students who consumed alcohol, more than half (n = 98) did so 2–4 times a month. The amount consumed per occasion was at least 3–4 drinks in a third of cases (n = 32), with 3% (n = 3) consuming more than 5 drinks. In addition, 1.3% of the total number of students (n = 4) reported using psychoactive substances, among which they indicated cannabis and marijuana.

### 3.3. Adherence to the Mediterranean Diet and Its Relationship with Sociodemographic Characteristics, Lifestyle, and Health Conditions

Overall, the mean MD adherence score was 7.64 (±1.98) points, with a range of 1–13 points. Based on the level of the adherence score, 35.6% of the students (n *=* 103) showed good adherence to the MD (score ≥ 9). In relation to each of the typical components of the MD, the consumption of “pieces of fruit” (21.8%), “portions of legumes” (33.2%), and “fish and seafood rations” (21.5%) were the items with the lowest adherence to recommendations (see Table 2).

**Figure 1:**
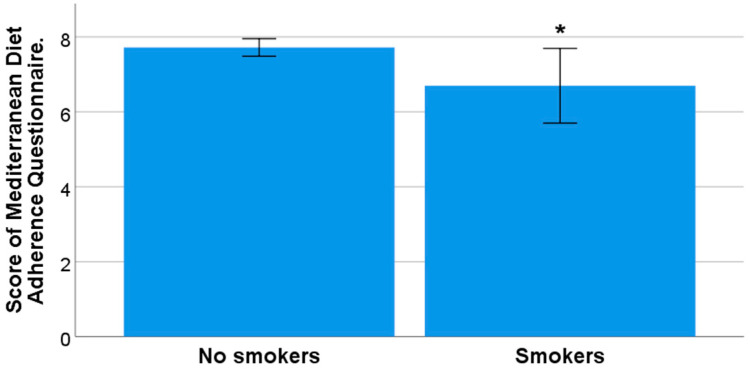
Mean score of the MD adherence questionnaire in smokers and non-smokers (* significant difference, *p* < 0.05).

No significant relationships were observed between recommended adherence to the MD (score ≥ 9) and gender (*p* = 0.140), marital status (*p =* 0.828), having children (*p* = 0.140), or place of residence during the academic year (*p* = 0.377). However, we observed a trend toward greater adherence to the MD as age increased (*p* = 0.057). No greater adherence to the MD was observed in those who consumed alcohol or psychoactive substances compared to those who did not (*p* = 0.597 and *p* = 0.546, respectively). Adherence to the MD was also not significantly associated with the consumption of stimulant drinks (*p* = 0.679) or with their frequency of consumption (*p* > 0.05 for coffee, tea, and cola). However, an association was observed between adherence to the MD and smoking (*p* = 0.038), with smokers showing significantly lower mean scores on the MD adherence questionnaire versus non-smokers (7.72 (±1.94) and 7.5 (±2.30), respectively) (Figure 1).

Adherence to the MD was also not associated with the different BMI categories (*p* = 0.849) or with being overweight/obese pooled together (underweight/normal weight versus overweight/obese) (*p* = 0.828), the presence of chronic disease (*p* = 0.191), or the daily consumption of psychotropic drugs (*p* = 0.358). However, although self-perceived health status did not show significant differences, we observed a trend toward greater adherence among those with a better self-perception of their personal health (*p* = 0.067).

### 3.4. Symptoms of Anxiety and Depression and Their Relationship with Sociodemographic Characteristics, Lifestyle, and Health Conditions

**Figure 2:**
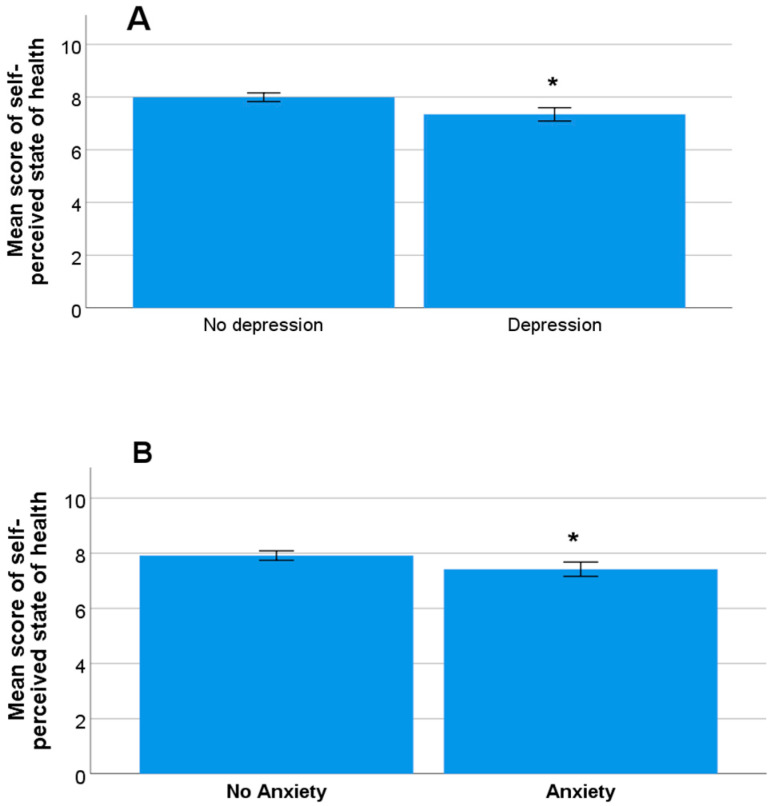
Mean self-perception of health status in students with or without symptoms of depression (**A**) and students with or without symptoms of anxiety (**B**) (* significant difference, *p* < 0.05).

As for the mood of the university students, the average score of the Goldberg total scale was 4.46 (±2.32) points (range 0–8), with mean scores for the anxiety and depression subscales of 2.83 (±1.34) points (range 0–4) and 1.64 (±1.35) points (range 0–4), respectively. With a cut-off point of 4 points or more in the anxiety subscale and 2 points or more in the depression subscale, 45.3% of the students (n = 131) were seen to present symptoms of anxiety, and 46.4% (n = 134) reported symptoms of depression. For the total score of the scale, on considering a value of 6 or more points, which is equivalent to the third quartile, 33.6% of the participants (n *=* 97) were seen to show symptoms of anxiety and depression. There was a statistically significant difference in the total score of the scale according to gender, with the average symptoms of anxiety and depression being higher among women than among men (4.59 (±0.52) versus 3.67 (±2.32); *p =* 0.017). As for the subscales, while gender did not show significant differences on the anxiety subscale (*p* = 0.104), we observed a trend toward depression in favor of women (*p =* 0.05). In relation to age, students with anxiety symptoms (anxiety subscale score ≥ 4 points) had a significantly higher mean age (20.84 (±2.46) years) than students with a score of <4 points on the anxiety subscale (20.41 (±2.65) years; *p =* 0.039). There were no significant associations between the age of the students and symptoms of depression (*p =* 0.399), nor did we observe significant differences in the mood of students in relation to marital status, having children, place of residence during the academic year, smoking, and the consumption of alcohol or stimulant drinks (*p* > 0.05 in all cases). Regarding the association of BMI with anxiety and depressive symptoms, in our study, there was no significant correlation between students’ BMI and total score on the Goldberg scale (*p =* 0.833). There was also no significant correlation between the total score of the Goldberg scale and BMI divided into four categories (underweight, normal weight, overweight, and obese) (*p =* 0.394). As for the subscales, there were also no significant differences between students’ BMI in relation to the anxiety subscale and depression subscale (*p =* 0.904 and *p =* 0.630, respectively). Separating BMI into three categories also showed no significant correlations with the Goldberg anxiety and depression subscales (*p =* 0.314 and *p =* 0.349, respectively). We found that students with a higher daily consumption of coffee presented greater symptoms of depression (*p* = 0.041), but not of anxiety (*p* = 0.307). Students with a depression subscale score of ≥2 points reported a poorer self-perception of their state of health compared to those with scores of <2 points (7.34 (±1.47) versus 7.99 (±1.02); *p* < 0.001) (Figure 2A). Likewise, those students with anxiety symptoms (anxiety subscale ≥4 points) had significantly lower mean scores in terms of self-perceived health compared to those who did not report anxiety symptoms (anxiety subscale < 4 points) (7.42 (±1.49) versus 7.92 (±1.07); *p =* 0.005) (Figure 2B). In turn, we observed that those students with greater symptoms of anxiety and/or depression reported poorer self-perceived health status (*p* < 0.001 for all scales). Lastly, while the consumption of psychotropic drugs on a daily basis was not associated with symptoms of anxiety (*p* = 0.301), the students who reported the daily use of psychotropic drugs had significantly higher mean scores than those who did not report such use on both the Goldberg total scale (5.85 (±2.30) versus 4.40 (±2.30)) and on the depression subscale (2.69 (±1.25) versus 1.58 (±1.34)) (*p* = 0.037 and *p* = 0.005, respectively).

### 3.5. Relationship between Adherence to the Mediterranean Diet and Students’ Mood

**Figure 3:**
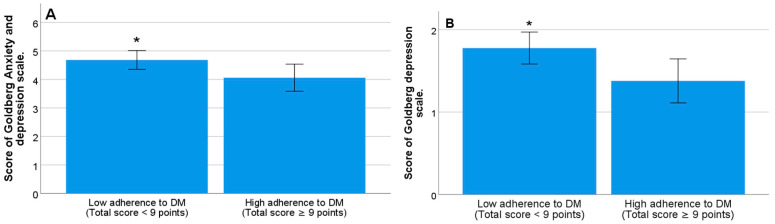
Mean score of the Goldberg total scale (**A**) and the depression subscale (**B**) in students with low adherence (score < 9) or high adherence (score ≥ 9) to MD (* significant difference, *p* < 0.05).

Adherence to the MD was associated with the mood of university students, with the proportion of students with depressive symptoms being significantly higher among those with low adherence to the MD (score < 9) (*p* = 0.005). Likewise, students with low adherence to the MD (score < 9), compared to those with good adherence (score ≥ 9), had significantly higher mean scores on the Goldberg total scale (4.06 ± (2.39) versus 4.68 (±2.25)) and on the depression subscale (1.38 (±1.36) versus 1.78 (±1.33)) (*p* = 0.039 and *p* = 0.013, respectively) (Figure 3A,B). Similarly, students who obtained higher scores on the Goldberg total scale and the depression subscale showed significantly lower scores on the MD adherence questionnaire (*p* = 0.011 and *p* = 0.004, respectively). Lastly, we observed a trend toward lower adherence to the MD among those who obtained a score of ≥2 points (*p* = 0.057) on the anxiety subscale.

### 3.6. Variables Associated with Low Adherence to MD: Logistic Regression Analysis

A multiple logistic regression model was applied to determine to what extent adherence to the MD was influenced by variables found to be related to low adherence to the MD (score < 9 points). Based on the variables that proved to be significant in the previous analyses, we analyzed smoking along with BMI, depressive symptoms, and the Goldberg total scale to try to explain and predict adherence to the MD in our study population. Thus, a backward statistical procedure was applied, with the initial model taking the multiple logistic regression model (which included the main effects of all explanatory variables) as input and including adherence to the MD as a response variable. Adherence to the MD was measured in terms of two categories: good adherence to the MD, with a score of ≥9 (category 0), versus low adherence to the MD, with a score of <9 (category 1). For categorical variables, the group that showed the best adherence in the previous analyses was chosen as the reference category. Regression model analysis indicated that the variables BMI, smoking, and the Goldberg total scale score did not significantly predict low adherence to the MD (score < 9). The final model selected the depression subscale score as a predictor variable, with the mean risk of low adherence to the MD being 0.803 times (95%CI: 0.666–0.968, *p* = 0.021) among the participants with greater depressive symptoms. Probability ratios, significances, and confidence intervals are presented in Table 3 below.

## 4. Discussion

College student mental health and wellness is a growing public health issue [40,41,42,43]. This study recorded a prevalence of depressive symptoms of 46.4% and a prevalence of anxiety symptoms of 45.3%. In Spain, mental health problems are highly prevalent in the general population [44], and according to a recent report by the Spanish Ministry of Health [15], the most frequent mental health problem is an anxiety disorder, which affects 6.7% of the general population (8.8% in women versus 4.5% in men). If “signs/symptoms of anxiety” are included, the figure rises to 10.4%. In our study, the prevalence of anxiety and depressive symptoms was higher than in previous research [45,46,47]. Although little is known about the mental health of university students, Ramón-Arbués et al. [45] conducted the first study on the prevalence of symptoms of anxiety, depression, and stress, and their associated factors, in a sample of Spanish university students. Compared with our study, these researchers found a lower prevalence of depression (18.4%) and anxiety (23.6%). The comparative increase could be due to the impact of the coronavirus disease pandemic (COVID-19) on the population studied. Indeed, the COVID-19 pandemic has affected the mental health of younger people, worsening their emotional well-being [48]. Young adults and adolescents have been especially vulnerable to the mental health consequences of the COVID-19 pandemic and lock-downs [49,50]. Another factor that contributed to the increase in anxiety and depressive symptoms among university students could be an increased prevalence of unwanted loneliness. Loneliness is an important risk factor for depression [49], and although the prevalence of loneliness varies with age, its association with depression remains stable across the lifespan [51,52,53]. The results of a recent study by the State Observatory of Unwanted Loneliness [54] claim that in Spain, 40% of people affected by unwanted loneliness are young individuals—a figure that doubles that recorded in people over 65 years of age. According to this study, 21.9% of people between 16 and 24 years of age suffer from unwanted loneliness. Most of the participants in our study were female nursing students (86.5% were females). The increased prevalence, therefore, could also be because depression is generally higher in women than in men [55,56], as in the case of depressive symptoms (but not anxiety symptoms) in our study, where the prevalence was found to be higher in female students than in male students. These results agree with those obtained by Ghrouz et al. [57], who stated that in at least two studies, female students had been shown to have poorer mental health compared to male students and were more likely to have depression and experience anxiety in university. Our findings are consistent with those of previous research, and we found a poorer self-perception of health status to be associated with depressive and/or anxiety symptoms [58,59]. Furthermore, in our study, the students who reported the daily use of psychotropic drugs, compared to those who did not consume such drugs, yielded significantly higher mean scores on the Goldberg total scale and the depression subscale. These data coincide with those of other studies, where a higher level of depression was found to result in increased consumption of psychotropic drugs [60] and should be considered for the adoption of other measures, such as nutritional interventions complementary to pharmacological treatment.

Regarding adherence to the MD—a gold standard of a healthy diet in southern European countries—only 35.6% of the students showed good adherence to the MD. These data coincide with those of other studies carried out in Spanish university students [61,62]. The findings are generally worrying for health, as it has been shown that mental health problems and poor adherence to the MD are associated in the general population [12,28]. According to the results of our study, university students show a significant correlation between adherence to the MD and depressive symptoms and the total score on the Goldberg scale. Thus, people with better adherence to the MD have fewer depressive symptoms and a lower total score on the Goldberg scale. In addition, we also observed a trend toward lower adherence to the MD among those students who had relevant anxiety symptoms (*p* = 0.057). Different studies have investigated the relationship between physical health and healthy habits. However, few studies have investigated the correlation between MD adherence and mental health, especially among college students [63]. To our knowledge, our study is the first to measure the relationship between adherence to the MD and mental health disorders (anxiety or depression problems) in university students. Sánchez-Villegas et al. [64] investigated the correlation between adherence to the MD and the incidence of clinical depression in a dynamic cohort of university graduates. They conducted a follow-up study involving 10,094 graduates of the University of Navarra (Spain) and the use of self-administered questionnaires and interviews. Higher adherence to the MD was significantly associated with a lower risk of depression. Another study by Zielińska et al. [16] in an adolescent population showed that the Mediterranean diet pattern can reduce the risk and symptoms of depression and improve rates of remission, while Western eating styles can increase the risk and severity of depression in adolescents. Two other studies [12,28] suggest significant benefits with the use of the MD in people with depressive symptoms, among which are a reduction in symptoms and better rates of remission. Specifically, Parletta et al. [12] investigated whether a Mediterranean-style diet supplemented with fish oil can improve mental health in adults suffering from depression. In addition, Jacka et al. [28] studied the efficacy of the Supporting the Modification of lifestyle In Lowered Emotional States (SMILES) program, based on the MD, with the inclusion of 67 subjects with Major Depressive Disorder. The diet intervention group demonstrated significantly greater improvement on the Montgomery–Åsberg Depression Rating Scale than the social support control group. Remission (defined as an MADRS score of <10) was recorded in 32.3% (intervention group) and 8.0% (control group) of the subjects, respectively. The review published by Antonopoulou et al. [63] evaluated adherence to the MD in different populations of university students. The authors found that higher adherence to the MD was correlated with a lower risk of depression. On the other hand, a higher perceived stress score was associated with a lower intake of fruits and vegetables. The students with greater adherence to the DM considered themselves to have better health, better quality of sleep, and a lower risk of depression. Additionally, higher fruit and vegetable consumption was associated with lower stress levels.

We observed an association between adherence to the MD and smoking habit. This finding is particularly interesting for preventive strategies because some studies have shown that low adherence to a Mediterranean diet pattern may exacerbate the negative health effects of smoking, exhibiting synergistic/multiplicative adverse effects [65]. 

Likewise, we observed a trend in favor of greater adherence to the MD among those with a better self-perception of personal health. Although, to date, no studies have revealed a relationship between adherence to the MD and self-perceived health, it has been shown that adherence to the MD is related to health-related quality of life, and in the case of women, there is also a relationship with life satisfaction [66].

Currently, the role of nutrition in mental health problems is considered more important than initially believed [67,68]. The protective effect of the MD against depression may be due to the combination of a sufficient intake of omega-3 fatty acids and other natural unsaturated fatty acids, together with antioxidants from olive oil and nuts, flavonoids and other phytochemicals from fruit and other plant foods, and large amounts of natural folates and other B vitamins [64]. Today, the increasing prevalence of depression and unhealthy lifestyles among young people requires new research to identify appropriate nutritional strategies capable of preventing and treating depression. Likewise, with early intervention, we can prevent the onset of depressive and anxious symptoms or the exacerbation of these symptoms. Consistent with our research results, prevention and intervention with an MD-based dietary pattern may be appropriate and cost-effective for reducing the rate of depression and anxiety in younger individuals. We could properly evaluate gender differences, as most of the nursing students are female students (13–15% of male students in our university in the last years). We cannot generalize our findings to all university students since nursing students could display differences compared to other degrees unrelated to health sciences. For instance, neither the consumption of alcohol nor psychoactive substances was associated with changes in adherence to the MD. Likewise, in relation to the results of previous studies carried out in Spain [69,70], the low percentage of students who reported consuming psychoactive substances is surprisingly low compared with other studies performed on university students. This fact could be explained by the better self-care skills of nursing students, which is one of the pillars of nursing studies [71]. Similarly, since social attitudes play a large role in drug availability and social reward [72], students living in a family nucleus, like most of our students, could have had lower rates of illegal drug use and alcohol abuse compared to students living alone or with mates [72]. Most of the students in our study lived primarily with their nuclear family. The social environment has a major influence on eating behavior, with parents and friends being two of the most important social influences on youth [73,74]. Studies such as that of Deliens et al. [75] noted that students who had family and friends who rarely consumed soft drinks or who had lived with strict family rules consumed fewer soft drinks/energy drinks. Other studies, such as Pearson et al. [76], stated that parents who ate a lot of fruits and vegetables were more likely to have children who consumed higher amounts of fruits and vegetables. Based on these results, it is interesting that preventive health education measures focused on dietary habits should include all the people living together in the family unit. Likewise, the approach to students with low adherence to the MD should be carried out from a socio-familial perspective. It should also be considered that other factors that influence the development of general eating patterns could be involved in good adherence to the DM, such as more health-promoting family eating patterns and attitudes, higher self-esteem, and a greater sense of coherence, which in turn could be related to increased eating competence, a flexible and positive approach to eating nutritious and enjoyable foods [77]. The link between eating competence and adherence to the DM in this population should be investigated in future studies; such understanding is important to ensure the development of health promotion programs. Likewise, the level of impulsiveness and meal participation could have influenced the dietary behavior of our university students [78]. The findings of our study should support the implementation of prevention and health promotion programs in universities that include the MD due to its positive effect in improving depressive and anxious symptoms in university students. In this regard, it would be interesting to adopt prevention policies and campaigns among university students to increase knowledge about healthy eating habits.

## Figures and Tables

**Table 1:** Characteristics of the study sample.

**Qualitative Variables**		**Frequency (n)**	**Percentage (%)**
Gender	Female	250	86.5
	Male	39	13.5
Nationality	Spanish	276	95.5
	Other	13	4.5
Marital status	Single	276	95.5
	Married	13	4.5
Children	No	288	99.7
	Yes	1	0.3
Cohabitation	Family	198	69.5
	Friends	67	23.2
	Couple	15	5.2
	Other	9	3.1
Chronic diseases	No	238	82.4
	Yes	51	17.6
BMI (kg/m^2^)	Underweight	30	10.4
	Normal weight	214	74.0
	Overweight	30	10.4
	Obese	11	3.8
Stimulating drinks daily (coffee, cola, tea)	No	110	39.1
	Yes	179	61.9
Tobacco	No	266	92
	Yes	23	8
Alcohol	No	121	41.9
	Yes	168	58.1
Psychoactive substances	No	285	98.6
	Yes	4	1.4
Psychotropic drugs daily	No	276	95.5
	Yes	13	4.5
**Quantitative variable**		**Mean (±SD)**	**Range**
Age (years)		20.60 (±2.56)	17–30
Self-perceived health (0–10)		7.69 (±1.29)	1–10

**Table 2:** Percentage of individuals who complied with the recommended frequency of consumption (weekly) of the foods included in the “MD adherence questionnaire”.

Items of the Questionnaire Corresponding to Good Adherence to MD	% Individuals over the Total Sample Who Fulfilled the Recommended Pattern of Consumption of Each Food
Use olive oil as the principal source of fat for cooking	92.7%
≥4 tablespoons of oil/day	41.2%
≥2 servings of vegetables/day	49.8%
≥3 servings of fruit/day	21.8%
<1 serving of red meat, hamburgers, sausage/day	68.2%
<1 serving of butter, margarine, cream/day	97.6%
<1 serving of carbonated sweetened beverages/day	90.3%
≥3 servings of legumes/week	33.2%
≥3 servings of fish or seafood/week	21.5%
<2 servings of commercial pastries/week	54.7%
≥3 servings of nuts/week	38.1%
Prefers white meat over red meat	83%
≥2 times/week homemade tomato sauce	71.3%
Individuals with good adherence to MD based on proposed cut-off value (total score ≥ 9 points)	35.6%

**Table 3:** Multiple logistic regression analysis: variables associated with low adherence to MD (score < 9).

Variables	*p*-Value	Exp (B)	95%CI EXP (B)	
			LL	UL
BMI	0.622	0.969	0.790	1.188
Smoking	0.198	0.508	0.181	1.424
Goldberg total scale score	0.761	1.019	0.947	1.096
Depression subscale score	0.021	0.803	0.666	0.968

LL: lower limit; UL upper limit. X^2^ Hosmer–Lemeshow = 3.642, Sig. = 0.303. “Exp(B),” is the odds ratio, is the predicted change in odds for a unit increase in the predictor. The “exp” refers to the exponential value of B.

## Data Availability

The data presented in this study are available on request for scientific purposes from the corresponding author.
